# Robust online learning based on siamese network for ship tracking

**DOI:** 10.1038/s41598-023-32561-0

**Published:** 2023-05-05

**Authors:** Zhongyi Hu, Jingjing Shao, Feiyan Nie, Zhenzhen Luo, Changzu Chen, Lei Xiao

**Affiliations:** 1 Intelligent Information Systems Institute, Wenzhou University, Wenzhou, 325035 China; 2 School of Intelligent Manufacturing and Electronic Engineering, Wenzhou University of Technology, Wenzhou, 325088 China

**Keywords:** Engineering, Mathematics and computing

## Abstract

The complex and changeable inland river scenes resulting out of frequent occlusions of ships in the available tracking methods are not accurate enough to estimate the motion state of the target ship leading to object tracking drift or even loss. In view of this, an attempt is made to propose a robust online learning ship tracking algorithm based on the Siamese network and the region proposal network. Firstly, the algorithm combines the off-line Siamese network classification score and the online classifier score for discriminative learning, and establishes an occlusion determination mechanism according to the classification the fusion score. When the target is in the occlusion state, the target template is not updated, and the global search mechanism is activated to relocate the target, thereby avoiding object tracking drift. Secondly, an efficient adaptive online update strategy, UpdateNet, is introduced to improve the template degradation in the tracking process. Finally, on comparing the state-of-the-art tracking algorithms on the inland river ship datasets, the experimental results of the proposed algorithm show strong robustness in occlusion scenarios with an accuracy and success rate of 56.8% and 57.2% respectively. Supportive source codes for this research are publicly available at https://github.com/Libra-jing/SiamOL.

## Introduction

The unprecedented growth in the inland river shipping industry resulted in social problems such as traffic congestion, frequent accidents, and serious environmental pollution threat. The safety management of the inland waterway operations in China needs urgent optimization. At present, the technical means applied to inland river ship monitoring mainly include automatic identification system (AIS)^1,2^ and radar tracking^3^. However, AIS has some problems such as insufficient function and improper information utilization. Radar tracking is easily disturbed by waves, coastal scenery and other factors, so there are certain blind areas. Therefore, as an auxiliary technical means, video surveillance helps to improve the intelligence level of inland river supervision and has a positive effect on the safe operation of inland river shipping.

In recent years, many researchers have used computer vision technology to analyze object information in videos to achieve ship detection and tracking. Xiao et al.^4^ designed a short-term tracker based on random projection under the framework of TLD^5^, which can significantly alleviate the tracking drift of ships under occlusion. Chen et al.^6^ proposed a framework integrating multi-view learning algorithms and sparse representation methods to extract highly coupled and robust ship descriptors from multiple different ship features. Since the target ship is often partially or completely occluded by marine obstacles, Chen et al.^7^ proposed an enhanced ship tracking framework through KCF^8^ and a curve-fitting algorithm. However, the above methods usually use hand-crafted features and lack generalization ability for challenging application scenarios. Recently, deep learning-based methods have shown promising performance in ship tracking. Shan et al.^9^ adopted an improved Siamese network combined with a multi-region proposal networks (RPNs)^10^ to build a maritime ship tracking framework. Yang et al.^11^ proposed an enhanced SiamMask^12^ for coastal ship tracking because of the lack of contour and edge information extracted from the Siamese network. Although Siamese network-based methods are widely applied in ground tracking, there are few studies on ship tracking, especially for inland river scenarios.

Moreover, the direct application of the Siamese network to inland river ship tracking has some deficiencies. Firstly, the Siamese network has poor discriminative ability to distinguish the object from the background when faced with the interference of similar objects. Secondly, the Siamese network cannot handle the object occlusion well, which may cause object tracking drift or even loss. Finally, the Siamese network usually updates the template per frame, but too frequent updates may introduce too much background information into the target model, resulting in template degradation, thereby increasing the probability of tracking drift. To deal with the above problems existing in the application of the Siamese network to inland river ship tracking, a robust online learning ship tracking method is proposed in present study. The main contributions are as follows:

- A weighted fusion method of off-line Siamese network classification scores and online classifier scores is proposed. Then an occlusion determination mechanism is established based on the classification fusion scores. When it is determined that the object is in the occlusion state, the object template is not updated. At the same time, a global search strategy is used to relocate the object, to avoid the object tracking drift.
- An efficient adaptive online update strategy, UpdateNet^13^, is introduced to improve the template degradation problem in the tracking process.
- On comparing the state-of-the-art tracking algorithms on the inland river ship dataset, our algorithm experimental results exhibit strong robustness in the occlusion scenarios.

## Related work

The research on general object tracking method based on computer vision not only has inspired but contributed valuable theoretical knowledge for the inland river ship tracking. Therefore, this section mainly introduces and comprehensively analyzes the status quo and development of visual object tracking, which will pave the way for the following research work. Due to the different ways of constructing object appearance models, visual tracking algorithms are generally divided into generative and discriminative.

The basic idea of the generative method is to model the object region in the current frame, and then find the most similar region to the model in the next frame, which is the predicted object position. Comaniciu et al.^14^ proposed a real-time tracking method, which can quickly find the most similar position to the target with fewer mean iterations. Bao et al.^15^ were the first to introduce sparse coding techniques into the L1 tracker of video image tracking, and developed a fast numerical solver based on the accelerated proximal gradient method to solve the 1-norm minimization problem and guarantee quadratic convergence. However, most trackers based on the sparse representation have high computational costs. Therefore, Zhang et al.^16^ efficiently solved the optimization problem by exploiting the circulant object template and Fourier transform, resulting in significantly improved tracking performance. Although the generative methods work well in majority of the above cases, most of the available methods only focus on the characteristics of the object and ignore its associated characteristics and the background or other non-objects, resulting in failure to track in extreme cases.

Compared with the generative methods, the discriminative is a classifier equipped with a learning function, and then the classifier is trained with the object, background, and other available information. Therefore, the discriminative methods perform better than the generative methods in terms of tracking effect. As a classic object tracking technique, the correlation filter tracker is favored by the researchers because of its fast speed. Bolme et al.^17^ proposed the MOSSE algorithm, which firstly introduced a correlation filter into the object tracking. Its high speed and good robustness make MOSSE more potential to solve the tracking problem. Subsequently, Henriques et al.^18^ proposed CSK and KCF successively to improve the accuracy of the correlation filter. CSK uses a cyclic structure for correlation detection of adjacent frames, while KCF uses the Gaussian kernel function to accelerate based on CSK, and extends single-channel grayscale features to multi-channel HOG features, which further improves the tracking accuracy. Since the above methods do not consider scale variation, the target scale is assumed to be unchanged in the whole tracking process, so it is not robust to the object with a sharp change in the scale. Based on this, Li et al.^19^ designed an efficient scale-adaptive method to solve the problem of fixed template size in kernel correlation filter tracker. To further improve the correlation filter algorithm, Li et al.^20^ suggested a new method for the boundary effect problem, which can handle the boundary effects without losing too much efficiency by combining temporal and spatial regularization. On this basis, Hu et al.^21^ presented a context-aware method that allows enlarging the object search region and add a part of the background to the object region. Then, by optimizing the regression objective function, the objective model is optimized according to the error minimization criterion. Whereas, Held et al.^22^ advocated a new network architecture and realized deep learning-based object tracking at 100 fps for the first time. As a result, deep learning has become an indispensable key technology in the field of visual tracking. Subsequent research focused mainly on developing off-line trackers to distinguish whether two images contain the same object by using a Siamese network.

However, on comparing the state-of-the-art correlation filter-based methods, the available Siamese network-based trackers (such as SINT^23^, SiamFC^24^, CFNet^25^, and SINT++^26^, etc.) methods are not satisfactory in terms of accuracy and speed. Realizing this, Li et al.^27^ applied the RPN module in object detection to the tracking task, and transformed the original similarity calculation into a regression and classification problem. The presence of a regression branch enables the algorithm to remove the original scale pyramid. Therefore, SiamRPN exhibits the state-of-the-art balance between accuracy and speed, and has great potential in more applications. Zhu et al.^28^ improved SiamRPN by adding semantic background and intra-class interference as negative samples to improve the recognition ability. They also proposed a distractor-aware module to reduce the response of the distractor and background, and adopted a local-to-global search strategy to re-detect objects when tracking fails. Wang et al.^12^ combined visual object tracking with video object segmentation for the first time, and proposed the SiamMask algorithm. It enables a fully-convolutional Siamese tracker to generate class-independent binary segmentation masks of target objects. Bhat et al.^29^ developed an end-to-end Siamese network tracking architecture that fully exploits both the object and the background appearance information for object model prediction. Building on this, Danelljan et al.^30^ proposed a probabilistic regression formulation and applied it to track. They predicted the conditional probability density of the object state given an input image through the network. However, all these trackers adopt off-line methods and lack the contextual information of the target ship, so they do not have sufficient discriminative ability to deal with distractors. Therefore, Yao et al.^31^ focused on the temporal smoothness between framesand proposed a two-stream network to learn discriminative spatio-temporal feature representations to represent the target objects. In addition, the discriminative ability of the conventional Siamese trackers is limited by the insufficient template-candidate representation. Yao et al.^32^ also proposed a Lucas-Kanade network (LKNet) and incorporated it to the Siamese architecture, which can dynamically transform template-candidate features to a more discriminative viewpoint for similarity matching. Since off-line ignore the target-background discriminative information, and lack the flexible target-specific update strategy, Fan et al.^33^ proposed an adaptive and discriminative Siamese complementary tracking network with flexible update mechanism. Inspired by the above Siamese tracking method, this paper proposes an online learning ship tracking algorithm based on the Siamese network. The algorithm combines the off-line Siamese network classification score and the online classifier score for discriminative learning. Moreover, an efficient adaptive online update strategy, UpdateNet, is introduced to improve the template degradation problem in the tracking process.

## Proposed algorithm

**Figure 1:**
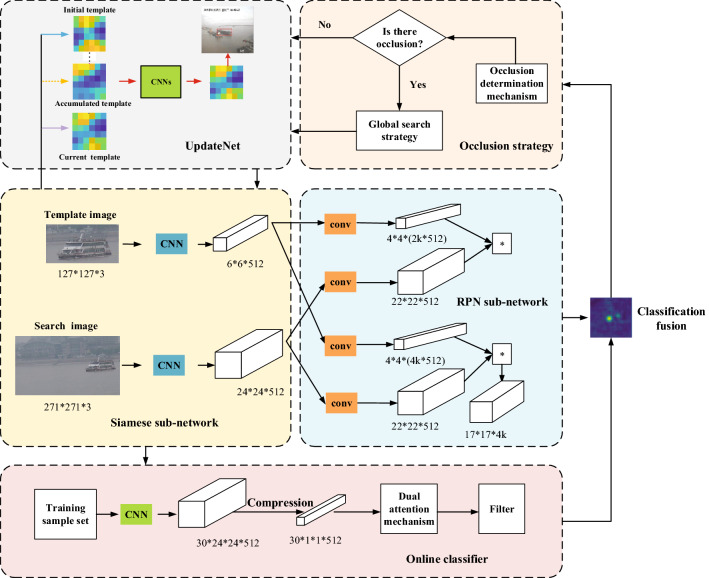
The architecture of SiamOL. The middle left is the Siamese sub-network for feature extraction. RPN sub-network lies in the middle right, which has two branches, one for classification and the other for regression. The top is UpdateNet for model update. The online classifier lies at the bottom, which consists of three parts: compression, dual attention mechanism, and filter. The classification fusion result is obtained by fusing the online classifier score map with the Siamese classification score map.

The tracking algorithm formulated in this research is Robust Online Learning Based on Siamese Network for Ship Tracking (SiamOL). The main architecture of SiamOL is shown in Fig. 1. SiamOL uses the algorithm model based on Siamese network. We first review the basics of Siamese network, including a description of classification branch similarity score. Next, we introduce the online classifier in ATOM^34^, and perform a weighted fusion of the output online classifier score with Siamese classification score. Finally, we propose a occlusion strategy, which uses the two-layer model updating network UpdateNet to learn “how to update” and the occlusion determination mechanism to judge “when to update”.

### Framework of SiamRPN

The Siamese network framework of SiamOL handles object tracking by using similarity learning. The similarity function uses the cross-correlation method by using the formula:

1 $$\begin{aligned} f(z, x)=\varphi (z)^{*} \varphi (x)+b_{1} \end{aligned}$$

Where, $\varphi$ is equivalent to the feature extractor, *z* is the template image, *x* is the image to be searched, $\varphi (z)$ is regarded as a convolution kernel, the similarity score is obtained by convolution on $\varphi (x)$, and $b_{1}$ represents the value of each position in the score map.

SiamOL is performed on the SiamRPN framework, which consists of Siamese sub-network and RPN sub-network. The Siamese sub-network uses the AlexNet^35^ to extract features, and the RPN sub-network consists of two branches: one is the classification branch, which is used for for foreground-background classification, and the other is the regression branch, which is used to obtain more accurate bounding box prediction. The classification branch gives the scores for each candidate region predicted as object and background, and performs the same transformation $\varphi$ on the two inputs *z* and *x* to generate the classification response map $f_{cls}(z, x)=[\varphi (x)]_{cls} *[\varphi (z)]_{cls}$. The regression branch is similar to the classification branch, but it gives the location regression value $f_{reg}(z, x)=[\varphi (x)]_{reg} *[\varphi (z)]_{reg}$ for each candidate region.

Since Siamese trackers lack contextual information for specific ship objects, they do not have sufficient discriminative ability to deal with distractors. Therefore, we make some improvements in classification branch, and add an online learning classifier to improve the discrimination ability of the tracking algorithm to distinguish between object and background.

### Classification fusion

We introduce an online classifier from ATOM, which is completely learned during online tracking and has sufficient discriminative ability to handle distractors. It is specially trained to predict the confidence of objects from the backbone features extracted from the current frame thereby, distinguishing objects from others in the scene.

The online classifier mainly includes compression module, attention module and filter module. The compression module adopts the random projection method to reduce the dimension, which can realize efficient real-time calculation. In addition, in order to solve the problem of unbalanced foreground and background samples, attention mechanism is used to capture rich semantic information, and dual attention mechanism is introduced to fully extract the characteristics of specific objects. The dual attention mechanism is the channel attention composed of two fully connected layers after global average pooling, and the spatial attention composed of a softmax after channel averaging. Finally, the filter module is used to generate classification confidence. Therefore, according to the above, the online classifier can be defined as follows:

2 $$\begin{aligned} f_{C}(x, w)=F(A(C(\varphi (x);w))) \end{aligned}$$

Where *w* represents the online learning network parameters, *C* represents the compression module, *A* represents the attention module, and *F* represents the filter module. Inspired by recent Discriminative Correlation Filters (DCF) methods, the objective function of similarity learning is defined in a form similar to L2 classification error:

3 $$\begin{aligned} L(w)=\sum _{i=1}^{m} \gamma _{i}\left\| f\left( x_{i} ; w\right) -y_{i}\right\| ^{2}+\sum _{k} \lambda _{k}\left\| w_{k}\right\| ^{2} \end{aligned}$$

The influence of each training sample $x_{i}$ is controlled by the weight $\gamma _{i}$. $y_{i} \in R^{W*H}$ is the annotated label, which is labelled with Gaussian function centered on the predicted object location. The penalty of regularization on $w_{k}$ is set by $\lambda _{k}$. For online classifier optimization, the Gauss-Newton descent method is used to solve quadratic problems.

We adopt a linear weighted fusion method to fuse the classification score map generated by off-line Siamese network and the score map generated by online classifier, so as to better distinguish the object from the background. Due to the lack of generalization to the learned model, online only methods are poor in object regression, while off-line only methods (such as fully convolutional Siamese trackers) lack target-specific contextual information and do not have sufficient discriminative ability to deal with distractors. Therefore, an online module with attention mechanism is proposed for off-line Siamese network to extract target-specific features under L2 error. The online classification score map is transformed into the same space size as the Siamese classification score map through bicubic interpolation, and then fused with a certain weight to obtain the adaptive classification score:

4 $$\begin{aligned} f_{fusion}(x, w)=\lambda f_{C}(x; w)+(1-\lambda ) f_{cls}(z, x ; w) \end{aligned}$$

Where $\lambda$ is the impact factor of the confidence score.

### Occlusion strategy

In the practical application of inland shipping supervision, there are many factors that affect the performance of ship tracking algorithm, among which occlusion occurs frequently. There are some deficiencies in the application of Siamese network to ship tracking. Siamese network cannot deal with the problem when the target is severely occluded, and it is easy to cause the target to drift or even be lost. To make our tracker solve the occlusion problem more effectively, a occlusion strategy is adopted in this section to judge whether the target is in an occlusion state. When the target is determined to be in an occluded state, the model is not updated, and a global search strategy is used to relocate the target to improve the overall performance of the tracker.

#### Occlusion determination mechanism

In order to solve the occlusion problem more effectively, the occlusion determination mechanism^36^ is used to improve the overall performance of the tracker. The occlusion problem usually has two characteristics: phased and gradient. The occlusion process is mainly divided into three stages: occlusion start, occlusion maintenance and occlusion detachment.

**Figure 2:**
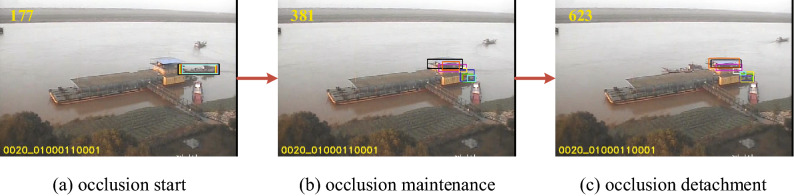
An overview of occlusion phases. (**a**) This is occlusion start phase, indicating that the object has just entered the occlusion state. (**b**) This is occlusion maintenance phase, indicating that the object is in the occlusion state. (**c**) This is occlusion detachment phase, indicating that the object has moved away from the occluder.

Figure 2 describes the process of a ship entering the occlusion, being occluded by a stationary occluder, and finally getting out of occlusion. Figure 2a depicts that the object bounding box is locked to a moving ship at the beginning, but when the video sequence runs to the frame of Fig. 2b, the stationary occluder blocks the moving ship. At this time, since the tracking algorithm cannot obtain the correct object model, but incorrectly extracts the model information of the occluder, the tracking algorithm mistakenly thinks that the occluder is the object model. In subsequent frames, the occluder is continuously tracked, and the wrong tracking object is not corrected in time, resulting in a “drift” phenomenon as seen in Fig. 2c, which leads to the failure of object tracking.

The occlusion process shows a gradient of a moving ship being occluded by a stationary occluder as depicted in Fig. 2a–c, which is a gradual process without any abrupt changes.

The phased characteristics of occlusion are used since the tracking algorithm extracts the features of the occluder during occlusion, the “drift” phenomenon occur in object tracking. Therefore, in the occlusion process, the object model of the frame before the occlusion starts is used and in the subsequent frames this correct object model is used for model matching. So to determine which frame is the occlusion start frame is the key to research. Since the occlusion process is gradual, the classification score will inevitably decline continuously. When the difference between the initial frame and the current frame during the decline is greater than the set threshold, it can be determined that occlusion has started. Similarly, at the end of the occlusion, it is symmetrical with the process of the beginning of the occlusion, and the classification score gradually increases. When the difference between the classification scores is greater than the set threshold it indicates that the occlusion is over. According to the change in degree of classification score, we propose an adaptive occlusion determination mechanism. The current state can be evaluated against the classification fusion score to decide whether the model should be updated and the search region should be expanded.

If $Z_{k}$ represents the tracking state in the *k*-th frame, $Z_{k}=0$ represents a stable state, which means that the tracking performance is stable. $Z_{k}=1$ represents an occlusion state, which means that the tracking performance deteriorates and the tracker tries to recover the performance. Furthermore, we also assume three thresholds (high threshold $t_{h}$, low threshold $t_{l}$ and tolerance *tol*). For the first frame, $Z_{k}$ is set to zero, $S_{k}$ represents the highest classification score in the *k*-th frame, and $\Delta S_{k}=S_{k}-S_{k-1}$. For the *k*-th frame, the state transition of occlusion determination mechanism is shown in Table 1.

**Table 1:** The state transition table of occlusion determination mechanism.

Condition	$Z_{k+1}$	The *k*th frame condition
1	0	$S_{k}>0$, $\Delta S_{k}>0$, $Z_{k}=0$
2	0	$S_{k}>t_{h}$, $\Delta S_{k}>0$, $Z_{k}=1$
3	0	$S_{k}>0$, $\Delta S_{k}<0$, $Z_{k}=0$, $\left| \Delta S_{k}\right| <tol$
4	1	$S_{k}<t_{h}$, $\Delta S_{k}>0$, $Z_{k}=1$
5	1	$\Delta S_{k}<0$, $Z_{k}=1$
6	1	$\Delta S_{k}<0$, $\left| \Delta S_{k}\right| >tol$
7	1	$S_{k}<t_{l}$, $\Delta S_{k}>0$, $Z_{k}=1$

According to the classification score change degree and tracking state of the current frame, the tracking state of the next frame is predicted.

In Table 1, in conditions 1–3, the tracker will update the model in the next frame. The meaning of these conditions can be summarized as follows: if the current tracker state remains stable, we should consider the current classification score $S_{k}$ and the classification score difference $\Delta S_{k}$. If they are all greater than zero, the tracker is considered to be performing well. Even if, $\Delta S_{k}<0$, the change degree of classification score is less than *tol*, there is no need to stop updating the model. If the current frame is occluded, the model cannot be updated in the next frame until the classification score is large enough, otherwise the model still fails to update, as shown in condition 4. For conditions 5–7, the model stops updating in the next frame. The meaning of these conditions is that if it is currently occluded, but $\Delta S_{k}<0$, the model cannot be updated in the next frame. If the classification score changes more than *tol*, the model cannot be updated. However, if the classification score drops significantly, we should prevent it from updating in the next frame.

The strategy is effective for tracking in occlusion situations and the main idea is to predict the occurrence of occlusion, stop the corresponding model update, and expand the search region. However, the tracking performance during occlusion cannot be guaranteed. In order to accurately locate the target, after predicting when to start occlusion, it is necessary to re-detect the target position after getting out of the occlusion (the object location during occlusion is not considered). The occlusion re-detection method first needs to determine whether the object begins to enter the occlusion state. When it is occluded, stop updating the template to avoid other background information polluting the template. After breaking away from the occlusion, re-detect the location of the object.

The re-detection method adopts a global search strategy. When the target is occluded for a long time and the tracking fails, the re-detection module can detect the target again, so that the tracker can recover from the error. Time is continuously tracked. The global search strategy adopts the traditional sliding window method: first, slide the input image from left to right and from top to bottom with sliding windows of different window sizes. Perform a template matching operation on the current window every time you slide. An object is considered to be detected if the current window gets a high similarity score. After each sliding window of different window sizes is detected, the object labels detected by different windows are obtained with have high repetition parts, and finally Non-Maximum Suppression (NMS) is used for screening. Finally, the detected objects are obtained after NMS screening.

#### Model update

**Figure 3:**
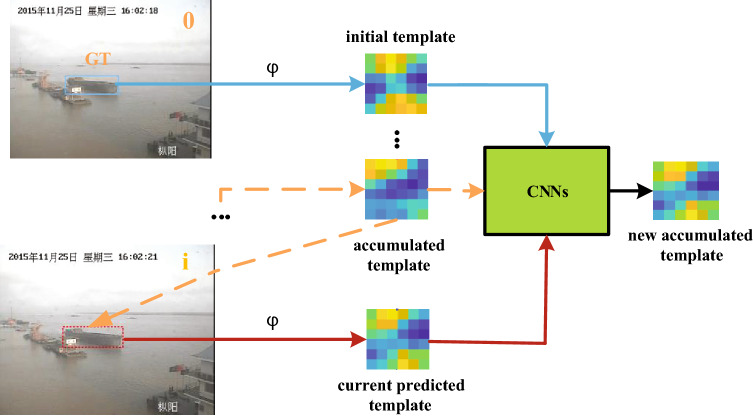
The framework of model update network. The online update of the object template adopts UpdateNet, which takes the initial ground-truth template, the accumulated template and the current predicted template as input, and outputs the new accumulated template.

In general, the model update method works by extracting an appearance template from the current frame, which is used to locate the object in the next frame. However, the linear combination of object templates will cause the object information to decay exponentially with time and thus, templates will degenerate. Although this update method broadens the improvement idea of tracking algorithms, its simplicity also limits the potential gains from learning to update. Therefore, we introduce an online learning update method, UpdateNet, which is obtained by training a two-layer Convolutional Neural Network (CNN). Given the initial template, the accumulated template and the template of the current frame, we directly use the trained UpdateNet model to estimate the optimal template of the next frame online , as shown in Fig. 3.

In order to solve the limitation of linear template update, we use a general learning function $\Phi$ to update the template, which is actually a CNN model, which has strong feature expression ability and the ability to learn from a large volume of data. The formula of $\Phi$ is as follows:

5 $$\begin{aligned} \dot{T}_{i+1}=\Phi \left( T_{0}^{GT}, \dot{T}_{i}, T_{i}\right) \end{aligned}$$

The learning function $\Phi$ is to calculate the updated template $\dot{T}_{i+1}$ given the ground-truth (GT) template $T_{0}^{GT}$ of the initial frame, the accumulated template $\dot{T}_{i}$ of historical frames, and the template $T_{i}$ extracted from the predicted target position in the current frame. Essentially, this function updates the previously accumulated template $\dot{T}_{i}$ by incorporating new information from the current frame $T_{i}$. Therefore, $\Phi$ can be adapted to the specific update requirements of the current tracking state according to the difference between the current template and the accumulated template. Furthermore, since the initial frame has highly reliable object information, the initial frame template $T_{0}^{GT}$ is considered, which improves the robustness to model drift.

As shown in Fig. 3, $T_{0}^{GT}$ is extracted from the object position GT in the initial frame (as number “0” in Fig. 3). To obtain $T_{i}$ of the current frame, the accumulated template $\dot{T}_{i}$ of the previous frame is used to predict the location of the object in *i*th frame (yellow dashed line), and the feature is extracted from this region (red solid line). The extracted initial template $T_{0}^{GT}$, current frame template $T_{i}$ and historical accumulated template $T_{i}$ are taken as new inputs, and then processed through a series of convolutional layers, and the predicted new accumulated template $\dot{T}_{i+1}$ is output. For the first frame, both $T_{i}$ and $\dot{T}_{i}$ are set to $T_{0}^{GT}$ since there is no previous frame. The only real information used by the model update network is the position of a given target in the initial frame, and all the other inputs are based on prediction.

## Experimental results and analysis

We adopt the evaluation method of OTB^37^ to comprehensively evaluate the performance of tracking algorithm. The OTB evaluation method mainly evaluates the success rate (i.e. center location error) and the precision (i.e. overlap rate) of the tracking algorithm, and then generates the corresponding success plots and precision plots, which can better observe the robust performance of the tracking algorithm. We apply the popular tracking algorithms in recent years to this framework, and compare the performance with our algorithm to verify the advanced and robustness of SiamOL. The experimental evaluations is performed on the inland river ship dataset and compared with seven state-of-the-art trackers namely, SiamFC, SiamRPN, DaSiamRPN, UpdateNet, Dimp, PrDimp, SiamMask.

**Figure 4:**
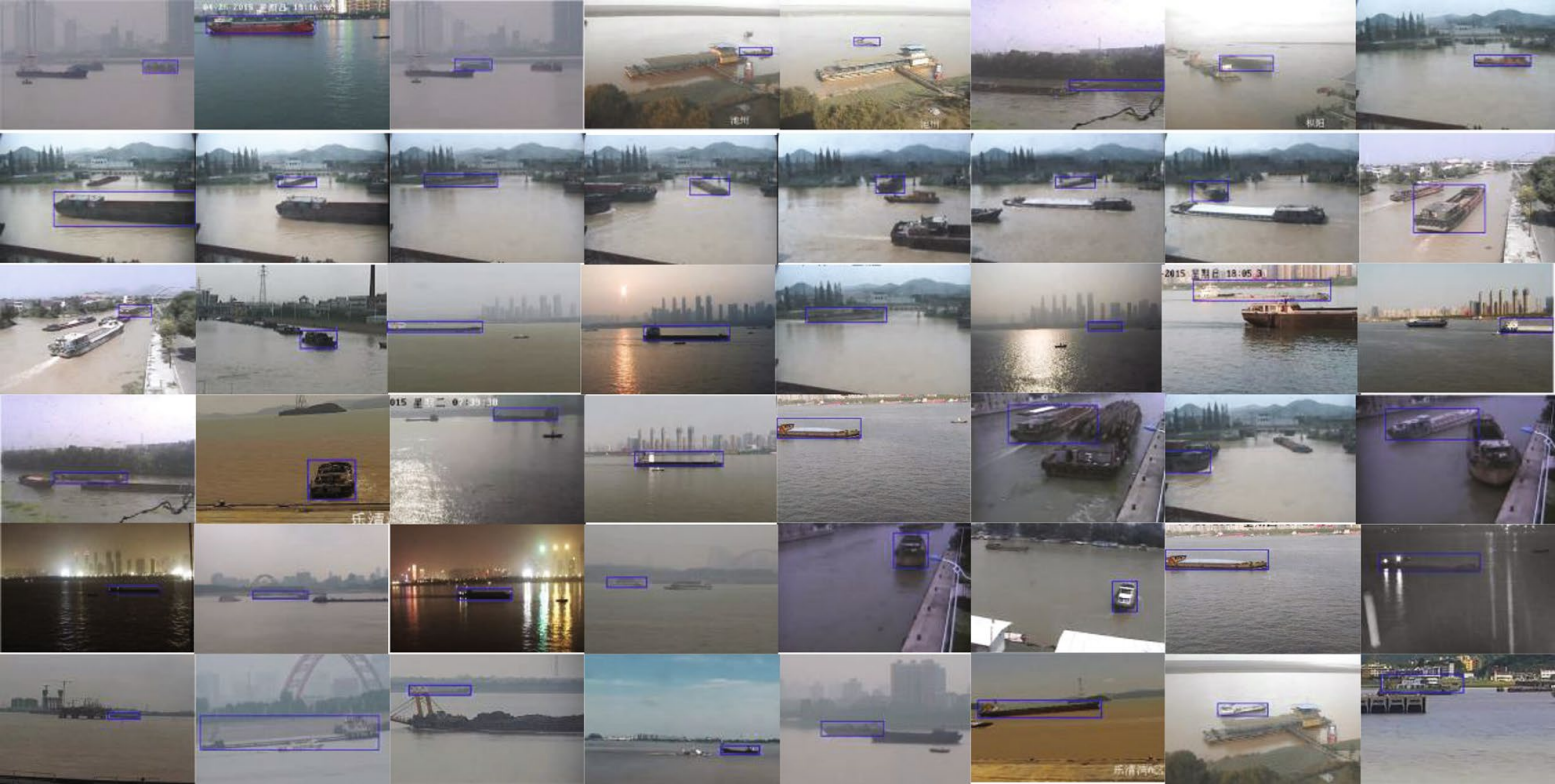
The inland river ship dataset. Excerpts of some manually annotated ship video images and the blue rectangle is the location of the object ship.

### Inland river ship dataset

We mainly conduct object tracking algorithm research on the inland river ship dataset, which is suitable for the practical application of inland waterway safety supervision. In order to establish an experimental platform for ship tracking and evaluation, we have collected some on the spot port ship videos. Based on the annotation and classification methods of OTB, the video is cut into picture frames; the location and size of ships are manually labeled by a rectangular frame, and classified according to different video attributes. There are 220 video sequences in the manual labeling ship dataset. As shown in Fig. 4, only part of the manually labeled ship video images is shown, and the blue rectangle in the figure is the object ship location.

### Implementation details

The experimental environment of the algorithm is PyTorch+Python3.7. All the experiments are completed on a desktop computer equipped with an Intel Core i5-9400 CPU at 2.90 GHz.

#### Training phase

We conduct experiments based on SiamRPN hence, directly used the official SiamRPN training model, and the entire tracking algorithm process does not require additional off-line training. The online training classifier also adopts Siamese network architecture, and generates 30 initial training samples by data augmentation on the initial frame. The model update also directly uses the available update network model, and performs online template update from the second frame.

#### Tracking phase

The online classifier updates the filter by discarding frames with distractors or missing objects. The classifier is updated every 10 frames, the learning rate is set to 0.01. The learning rate is doubled when a distractor is detected. For classification fusion, we conduct ablation experiments. It is observed through a large number of experimental comparisons that when $\lambda$ is 0.75, the tracking effect is the best. The model update is done through the occlusion determination mechanism. The hyper-parameters $t_{h}$, $t_{l}$ and *tol* are set to 0.93, 0.92 and 0.02, respectively, and the sliding window matching threshold is set to 0.5.

### Ablation experiments

We performed an ablation study to demonstrate the impact of each component in SiamOL. In this section, the experimental data we use is inland river ship dataset, which is evaluated on 26 ship video sequences with occlusion video properties.

#### Classification fusion

The score response map of the online classifier is transformed into the same space size as the Siamese classification through bicubic interpolation, and then fused with a certain weight to obtain the adaptive classification score. Among them, the weight is the penalty factor $\lambda$, and the size is between 0 and 1. The ablation experiments are performed on penalty factors of different sizes, and the results are shown in Table 2. When $\lambda$ is 0, it means only the off-line Siamese classification score, and when $\lambda$ is 1, it means only the online classifier score. It can be seen from the table that when $\lambda$ is 0.9, the success rate and precision are optimal at this time.

**Table 2:** Success rate and precision of different penalty factors on the inland river ship dataset.

$\lambda$	0	0.2	0.4	0.5	0.6	0.7	0.8	0.9	1
Success rate	0.544	0.551	0.546	0.541	0.543	*0.566*	0.565	**0.572**	***0.571***
Precision	0.527	0.547	0.539	0.533	0.530	***0.565***	*0.562*	**0.568**	0.548

Bold, bolditalic, and italic fonts indicate the top-3, respectively.

**Figure 5:**
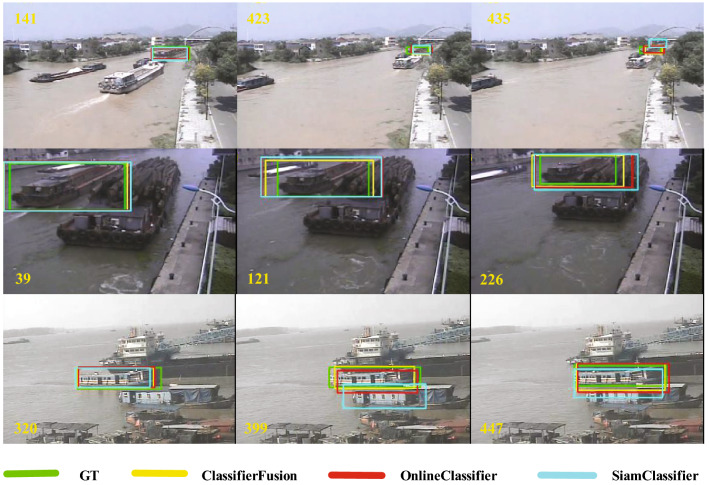
Qualitative evaluation of different penalty factors on three ship video sequences with occlusion attribute. ClassifierFusion (short for classifier fusion) is able to track more accurately than OnlineClassifier (short for online classifier) and SiamClassifier (short for Siamese classification) when the target is occluded.

Three representative video sequences are selected for the qualitative analysis, and the optimal classification fusion (ClassifierFusion) is compared with online-only classifier (OnlineClassifier), Siamese-only classification (SiamClassifier), and GT. As shown in Fig. 5, each row represents the same video sequence, and the three video sequences are all under occlusion interference. It can be seen that when the object is disturbed by occlusion, ClassifierFusion can show better performance and can accurately frame the location of the object.

#### Model update

The impact of the model update strategy is explored by comparing with the initial frame template update as well as the simple linear template update. The success rate and precision of the proposed model update method (Our), initial frame template update (WithoutUP) and linear template update (LinearUP) are shown in Table 3. It can be seen that the success rate and precision of our proposed template update method are 57.2% and 56.8%, respectively, and the tracking effect is the best in the case of occlusion. This is 5% and 4% higher than adding linear model updates, respectively.

**Table 3:** Performance comparison of different model update methods on the inland river ship dataset.

Tracker	Our	WithoutUP	LinearUP
Success rate	**0.572**	0.569	0.521
Precision	**0.568**	0.563	0.522

Bold font indicates the best.

### Experimental comparison

Our tracker SiamOL is compared with the state-of-the-art methods on the inland river ship dataset with occlusion video attribute. The dataset consists of 26 video sequences, and its performance is evaluated in terms of success rate and precision. Table 4 shows the comparison of our method with seven state-of-the-art trackers: SiamFC, SiamRPN, DaSiamRPN, UpdateNet, Dimp, PrDimp, SiamMask. Our algorithm achieves the best precision while having a competitive success rate.

**Figure 6:**
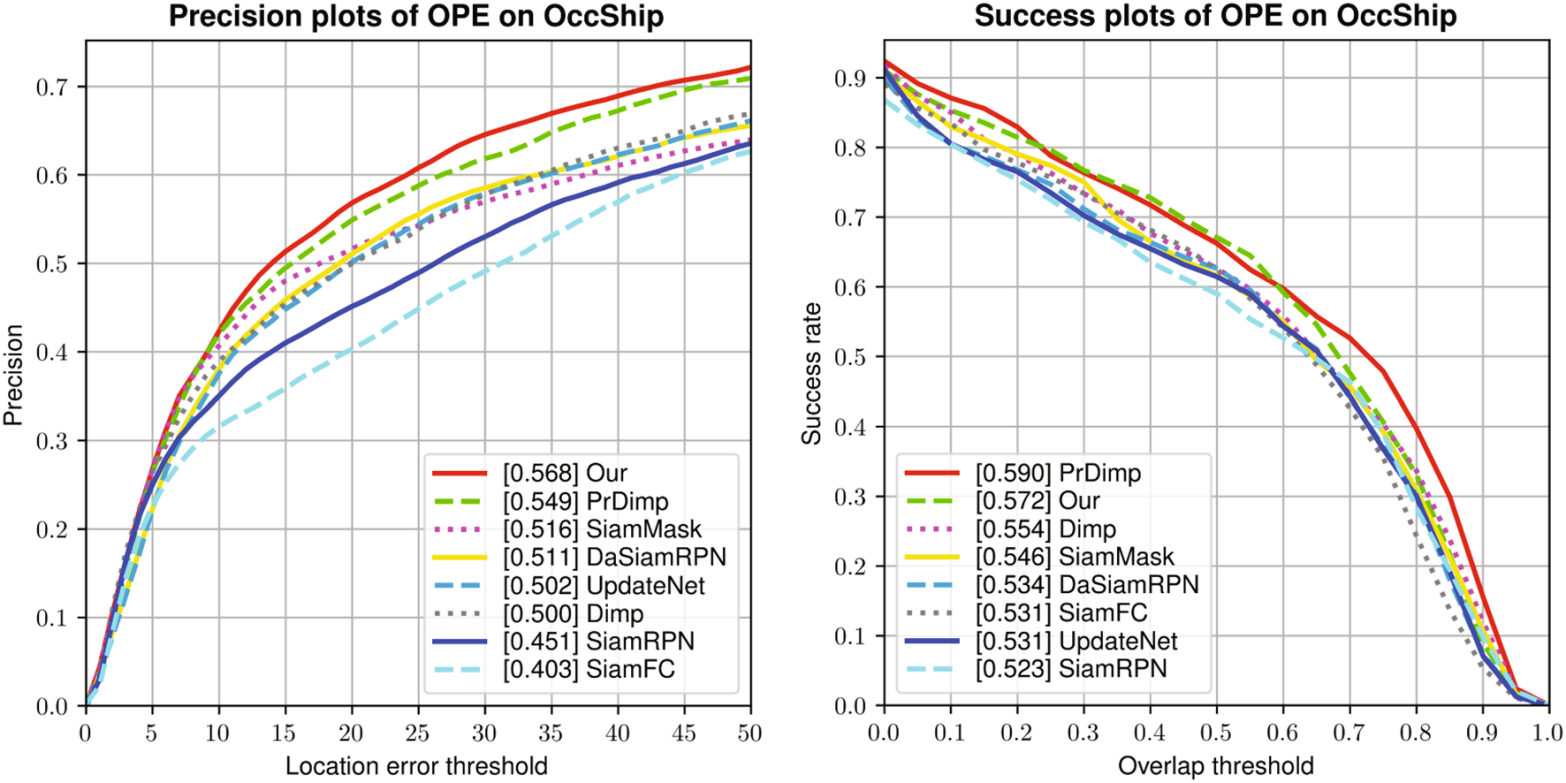
Performance evaluations of the inland river ship dataset in terms of success and precision plots. The legend contains the average distance precision score at 20 pixels and the area-under-the-curve (AUC) score for each tracker.

Furthermore, our tracker outperforms the suboptimal tracker by 2% in precision. Fig. 6 shows the precision plot and success plot corresponding to the trackers and the figure shows the average tracking performance of each video attribute. Since we test on the ship dataset with video attribute, it can be seen that the proposed algorithm performs well under the occlusion conditions.

**Table 4:** Comparison of 7 state-of-the-art trackers on the inland river ship dataset.

Tracker	Our	SiamFC	SiamRPN	DaSiamRPN	Dimp	PrDimp	UpdateNet	SiamMask
Success rate	***0.572***	0.531	0.523	0.534	*0.554*	**0.590**	0.531	0.546
Precision	**0.568**	0.403	0.451	0.511	0.500	***0.549***	0.502	*0.516*

Bold, bolditalic, and italic fonts indicate the top-3 trackers, respectively.

**Figure 7:**
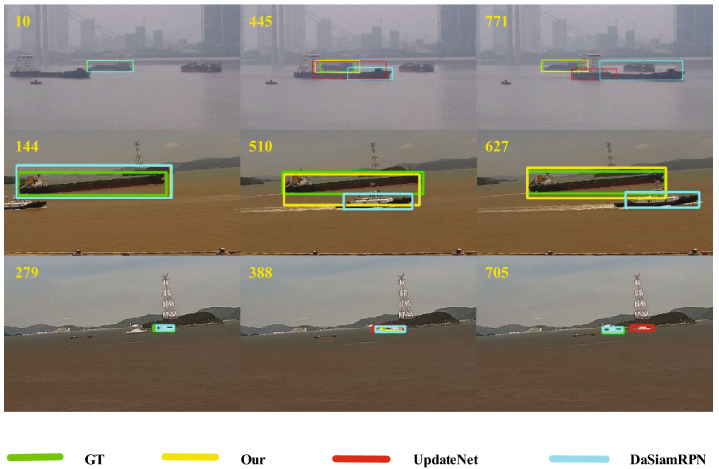
Qualitative evaluation of our tracker and two state-of-the-art trackers on the three ship video sequences with occlusion attribute. Our approach is able to track more accurately than UpdateNet and SiamRPN when the target is occluded.

Three representative video sequences are selected for qualitative analysis, and the proposed algorithm is compared with DaSiamRPN, UpdateNet and GT. As shown in Fig. 7, each row represents the same video sequence, and the three video sequences are all under occlusion interference. Each column represents different occlusion stages. The first column represents pre-occlusion stage, the second column represents occlusion maintenance stage, and the third column represents occlusion detachment stage. The first and the third video sequences are in the case of large-area occlusion. In the first video sequence, only our algorithm can continue to track the object accurately when the occlusion is removed, whereas the other trackers lose tracking. In the third video sequence, our algorithm and DaSiamRPN algorithm can continue to track the object accurately when the occlusion is removed. The second video sequence is in the case of small-area occlusion, only our algorithm can continue to accurately track the object, while other algorithms result in tracking the object “drift” due to the interference of the occluder, and then keep tracking the occluder. Our algorithm determines the object state through the occlusion determination mechanism, regardless of the tracking performance during the occlusion process, and uses the global search method to relocate the object after the occlusion is removed. Therefore, our algorithm shows better performance in the occlusion situation.

## Conclusion

In this paper, we propose a robust online learning ship tracking algorithm to improve the object tracking drift problems when ships are occluded by each other. Based on SiamRPN, the algorithm fuses the off-line Siamese network classification score and the online classifier score for discriminative learning, and establishes an occlusion determination mechanism according to the classification fusion score. When it is determined that the object is in the occlusion state, the object template is not updated, and the global search mechanism is activated to relocate the object, thereby avoiding the object tracking drift. In addition, an efficient adaptive online update strategy, UpdateNet, is introduced to improve template degradation problems during tracking. The experimental comparison with the state-of-the-art tracking algorithm on the inland river ship dataset shows that our algorithm exhibits strong robustness in the occlusion scenarios. However, our algorithm can only perform well when ships occlude each other, and needs to be further improved in other occlusion scenarios.

## Data Availability

All data generated or analysed during this study are included in the main text. In addition, the datasets used and/or analyzed during the current study are available from the correspondin g author on reasonable request.
